# Hospital admissions due to snake envenomation in the Republic of Cyprus: a 7-year retrospective review

**DOI:** 10.1186/s12995-022-00363-1

**Published:** 2022-12-21

**Authors:** Daniel Jestrzemski, Maria Athanasiadou, Vasos Scoutellas, Parviz Ghezellou, Bernhard Spengler, Frank Gessler, Ulrich Kuch

**Affiliations:** 1 Faculty of Forest Sciences and Forest Ecology, Department of Forest Zoology and Forest Conservation, University of Göttingen, Göttingen, Germany; 2 Institute of Occupational Medicine, Social Medicine and Environmental Medicine, Goethe University, Frankfurt am Main, Germany; 3 Health Monitoring Unit, Ministry of Health, Nicosia, Republic of Cyprus; 4 Institute of Inorganic and Analytical Chemistry, Justus Liebig University Giessen, Giessen, Germany; 5 miprolab Mikrobiologische Diagnostik GmbH, Göttingen, Germany; 6 Institut für angewandte Biotechnologie der Tropen e.V., University of Göttingen, Göttingen, Germany

**Keywords:** Snake envenomation, Epidemiology, Hospital admissions, Cyprus, Paphos, Blunt-nosed viper

## Abstract

**Background:**

Snake envenomation is a major neglected tropical disease, lacking data in many countries including Cyprus, a Mediterranean island inhabited by the medically important blunt-nosed viper (*Macrovipera lebetina*). Reviewing the 2013–2019 period, we present first-time epidemiological snakebite data in the Republic of Cyprus.

**Methods:**

We obtained data on snake envenomation-related hospital admissions from the Ministry of Health, and population and rainfall data from the Statistical Service of Cyprus and Department of Meteorology websites. Human-viper conflict information was acquired from interviews with 12 representatives of Cypriot institutions.

**Results:**

Between 2013 and 2019, 288 snake envenomation cases were admitted to public hospitals, averaging 41 people annually. The minimum was 29 cases (2017) and the maximum was 58 (2015). Snake envenomation incidence increased from 4.55 per 100,000 population (2013) to 6.84 (2015), but remained low since 2017 (3.49 in 2019). Between 2000 and 2018, the deaths of one man (73 years), and indirectly, one woman (77 years), were related to snake envenomation. While 266 cases (92%) happened between April and October (the blunt-nosed viper activity period), most envenomations occurred in September (cumulative for 2013–2019), with 88 cases (31%). Snakebite incidence peaked in the 60–69 years age group (9.19 per 100,000 population), and was higher in males (6.85) than in females (2.82). Of all admitted patients, 242 (84%) were discharged within 4 days. Mean hospital stay duration was 2.65 days, with one case of 13 days. Most patients were admitted to the general hospitals in Paphos (51%), Limassol (30%) and Nicosia (11%), which provide secondary healthcare, with the last one providing tertiary healthcare.

**Conclusions:**

Snakebite-related deaths are very rare in the Republic of Cyprus. Most envenomation cases happened in late summer (September). Short hospital stays indicate mostly non-severe clinical courses. The hospital admission data suggest that snake envenomation risk is highest in Paphos district. The statistical data hint at males and middle- to older-aged people being at highest risk, whereas from our interview data we assume that outdoor workers are at higher risk than other occupational groups.

## Background

**Fig. 1:**
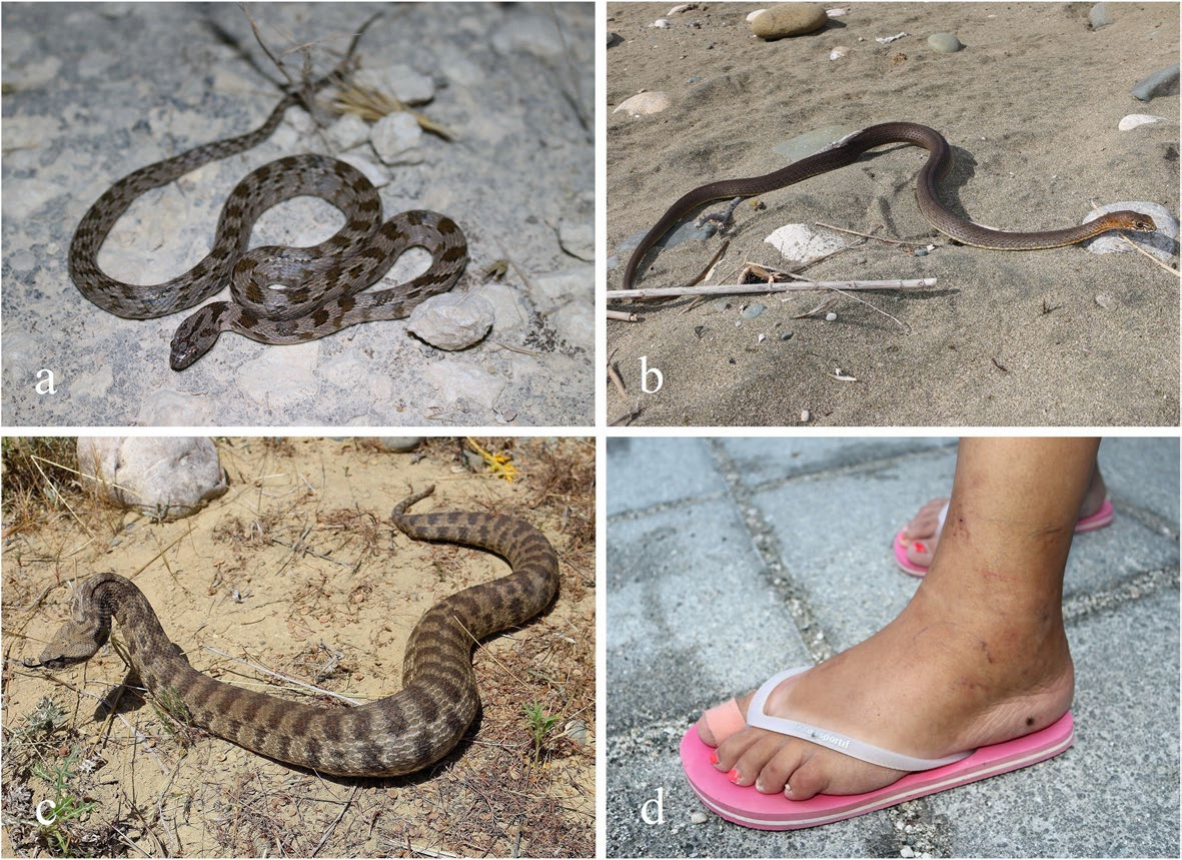
**a** Cat snake (*Telescopus fallax*), rear-fanged. **b** Eastern Montpellier snake (*Malpolon insignitus*), rear-fanged. **c** Blunt-nosed viper (*Macrovipera lebetina*), a medically important front-fanged species. **d** The swollen foot of a 35-year-old woman, 5 days after the bite by a juvenile *M. l. lebetina* in Latchi, Paphos district, Cyprus. Photos: D. Jestrzemski

Cyprus is the third largest Mediterranean island, measuring 224 km in length, 96 km in width and covering 9251 km^2^, of which 1733 km^2^ are forested. The distance to the nearest country is 75 km to Turkey in the north [1]. The island comprises three main geographic regions (Troodos Mountains, Keryneia Range and Mesaoria Plain) [1–3]. Cyprus has a subtropical Mediterranean climate with dry, hot summers and mild, humid winters (Köppen climate classifications Csa and BSh) [4]. It is the hottest and driest island of the Mediterranean, and has the most hours of sunshine. Annual precipitation varies from 300 to 350 mm (Mesaoria) up to 1200 mm (highest Troodos slopes), with the southwestern coastal districts receiving 450 mm. Due to its distinct dry and rainy seasons, with most rainfall occurring from November to March, Cyprus has no permanent rivers [2, 3]. Zoogeographically, Cyprus is considered part of Southwestern Asia. Three species of venomous snakes exist on the island: the cat snake (*Telescopus fallax*), the eastern Montpellier snake (*Malpolon insignitus*), and the blunt-nosed viper (*Macrovipera lebetina*) (Fig. 1). The cat snake (*T. fallax cyprianus*) is endemic to Cyprus at subspecies level, while the blunt-nosed viper is present on Cyprus with its nominate subspecies (*M. lebetina lebetina*) [2].

**Fig. 2:**
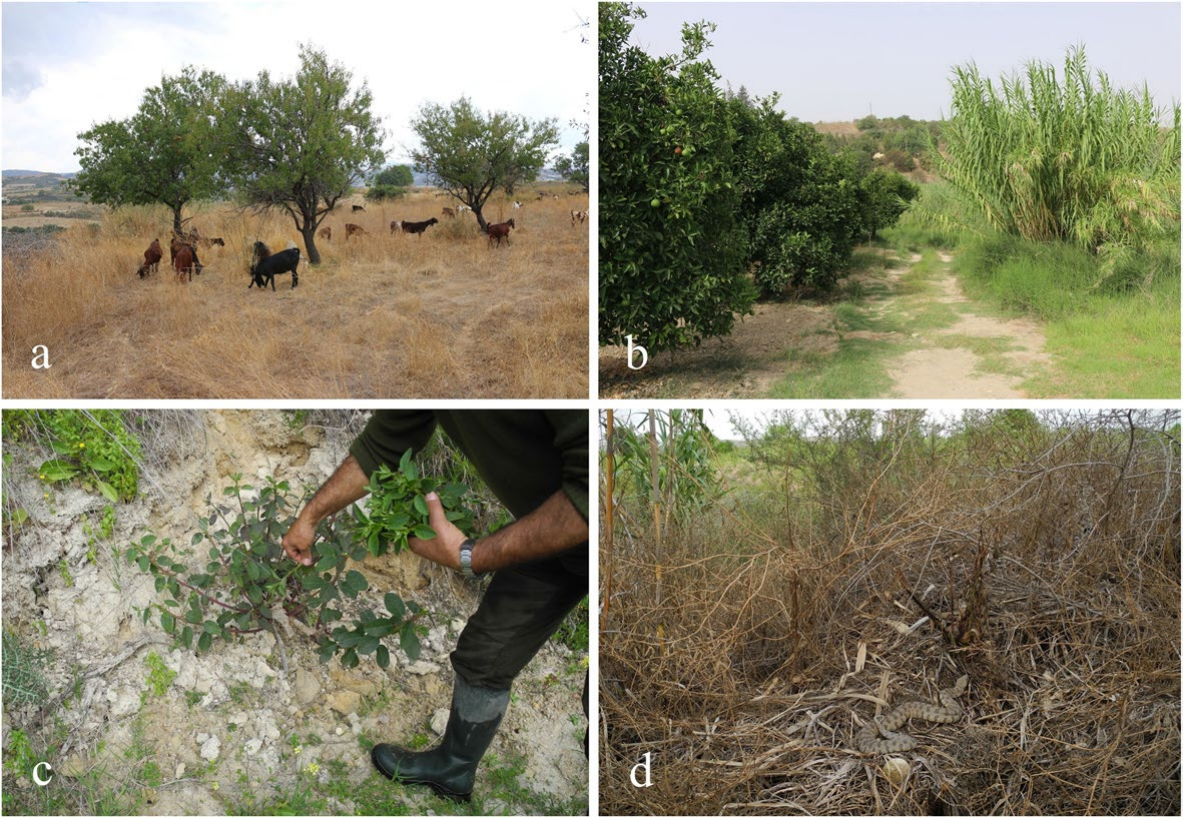
**a** Goat-grazing in *M. l. lebetina* habitat at the edge of farmland (19 September 2015). In Cyprus, snake envenomation is a constant risk to livestock. **b** Habitat of *M. l. lebetina* at the edge of an orange plantation and riparian vegetation, offering favorable conditions for rats and their ophidian predators (8 September 2015). **c** A local man harvesting capers (*Capparis spinosa*) (9 April 2014). As *M. l. lebetina* commonly ambushes prey underneath shrubs, including capers, agricultural workers in Cyprus face an increased risk of snake envenomation. **d** *Macrovipera l. lebetina* camouflaged at the edge of farmland and riparian vegetation (9 May 2014). Photos: D. Jestrzemski

While *T. fallax* and *M. insignitus* are rear-fanged venomous colubrids [5], *M. lebetina* is a large, medically important, front-fanged viper and a recognized cause of serious snake envenomation across its vast geographic distribution from Cyprus to Kashmir [6–9]. Although severe cases of envenomation by *M. lebetina* have been reported from the twentieth century, data of recent envenomation cases are scarce [8]. *Macrovipera l. lebetina* is relatively common in agricultural areas and rocky slopes (Fig. 2), occurring from the coastal lowlands to the Troodos mountains [2, 10]. Similarly, *M. insignitus* and *T. fallax* are found in stony habitats with well-developed vegetation (Phrygiana) [2]. While there are no documented cases of human envenomation by *T. fallax* and *M. insignitus* for Cyprus [2], 16–20 cases of snakebite by *M. l. lebetina* have been recorded annually on the island [11]. Bites to livestock have been reported as well [12]. Blunt-nosed viper venom mainly consists of the protein families Zn2+ − metalloproteinases (SVMPs, causing internal bleeding, intravascular clotting, edema, inflammation and necrosis), serine proteinases (SVSPs, impacting physiological functions such as blood coagulation, fibrino(geno)lysis and platelet aggregation), L-amino acid oxidases (LAAOs, inhibiting ADP or collagen-induced platelet aggregation), snake venom hyaluronidase (playing a crucial role in venom circulation and strongly contributing to the envenomation symptomology), phospholipases A2 (PLA2s, with neurotoxic, hemolytic, myotoxic, anticoagulant, antiplatelet and antibacterial effects), C-type lectin-like proteins (CLTs, biologically highly active and strongly affecting platelet aggregation) and disintegrins (strong inhibitors of platelet aggregation) [13]. Local symptoms of *M. lebetina* envenomation include local pain and swelling, bruising, lymphangitic markings, regional lymphadenopathy, necrosis and local blistering. Systemic symptoms comprise thirst, nausea, vomiting, trembling, hypotension, shock, tachycardia, syncope, cold sweating, cardiorespiratory distress and spontaneous bleeding [9]. In the Republic of Cyprus, antivenom administration is based on a routine indication procedure: If a patient reports to be bitten by a snake, the physician evaluates the bitten area for signs of inflammation, swelling and necrosis. If such signs exist, then antivenom is given, otherwise not. On the other hand, if the patient states that the bite was certainly delivered by a dangerously venomous snake species (of which only one exists in Cyprus), then this is judged by the physician in order to give the treatment, or not. Hence, a physician has to be sure that either 1) the snake was venomous, or 2) the clinical course of the snakebite shows a toxic effect on the patient. This is of importance since antivenom treatment might have serious side effects.

Snake envenomation is a cause of significant human morbidity around the globe and was recently ranked as a Category A Neglected Tropical Disease by the WHO [14]. According to Kasturiratne et al. (2008), worldwide 421,000 to 1,841,000 people are envenomed by snakes annually, of which 20,000 to 94,000 die [15]. Snakebite survivors often suffer from chronic morbidity and disability following envenomation, including amputations, blindness, contractures, chronic infections and malignant ulcers [16]. In addition to physical impairments, snakebite survivors commonly experience severe psychological trauma caused by the incident and its health consequences, which forces them to retire from work and causes the loss of the primary source of income for many families [17]. On a global scale, conservative estimates set the burden of snake envenomation at 6.07 million disability-adjusted human life years annually [18]. However, snakebite remains notoriously underreported [16]. As snakebite victims often face high treatment costs, enforced borrowing and loss of income, the disease is a significant factor exacerbating poverty [16]. Snakebites also cause damage to livestock, which adds to the economic hardship of poor rural communities [19]. In fact, snakebite primarily affects poor rural communities, who suffer from adverse living conditions that increase the likelihood of human-snake encounters, low income, and limited access to education and healthcare [20–23]. In Europe, snakebite represents a much lower medical emergency than in tropical developing countries [24], although several European viper species, including *Vipera berus*, *V. aspis* and *V. ammodytes*, may cause life-threatening envenomations [25–27]. Apart from vipers, the only other European snakes capable of causing serious, but usually not life-threatening envenomations are the rear-fanged Montpellier snakes in the genus *Malpolon* [5, 27]. From 1970 to 2010, the annual number of snakebite cases in Europe was estimated at 7992, with four deaths caused by snakebite per year on average. A better knowledge of snakebite epidemiology in Europe is necessary for an improved understanding of therapeutic requirements [24]. In the Eastern Mediterranean region (which includes the countries Greece, Cyprus, Turkey, Syria, Lebanon, Palestine, Israel, Jordan and Egypt) [28], several genera of medically highly important, venomous snake taxa occur, of which only *Macrovipera* (i.e., *M. l. lebetina*) is native to Cyprus. The annual number of snake envenomations in North Africa and the Middle East, with a total human population of about 160 million, is estimated between 3017 and 80,191, and between 43 and 78 deaths annually [15]. The available data on snakebite in the Middle East are highly fragmentary, and for some countries (e.g., Syria) no information is yet available [29]. Despite the medical importance of snake envenomation, data on its epidemiology is scarce for most countries, due to a lack of reliable snakebite reporting systems, as well as a low awareness of health authorities regarding the public health importance of snake envenomation. In addition, snakebite cases are often reported only internally in institutions or published in newspapers or local journals, precluding international scientific access [30]. The goal of this article is, therefore, to present first-time systematically obtained data on snakebite hospitals admissions in Cyprus, considering annual occurrence, sex and age distribution of the bite victims, and distribution of admissions among Cypriot public hospitals. In this publication, only the Republic of Cyprus (de facto the southern part of the island) is included, since no statistics are available on snake envenomation in the Turkish-controlled north part of Cyprus.

## Methods

We retrospectively analyzed data on public hospital admissions due to snake envenomations that were provided by the State Health Services Organisation of Cyprus to the Ministry of Health (Republic of Cyprus). Of all public hospitals, the percentage of coding was around 95% of all inpatients. No data were available for admissions in private hospitals. All snakebite-related admissions were based on the ICD-10 code T63.0 referring to “Contact with snake venom” [31]. For all discharges due to death from snakebite (within the premises of a hospital, as well as outside), a Medical Certificate for Causes of Death was completed and coded within the Health Monitoring Unit, as part of the national Registry of Causes of Deaths. All deaths records were based on coding with Iris software, an automatic system used for coding multiple causes of death, and for selecting the underlying cause [32]. From all seven public Cypriot hospitals, the sex (male or female), age group (in five-year-steps, from 0 to 89 years), length of stay (days spent in hospital) and monthly number of admitted snakebite patients were recorded for the years from 2013 to 2019. The data were provided in the form of two Microsoft Excel documents, containing tables and charts. The tables show the number of males and females envenomed by snakebite from 2013 to 2019, categorized by year and age group. This information is provided both for the overall number of snakebite incidents, and for each of the seven hospitals. Additional information for each hospital included the number of snakebite registrations per month and year, and the length of stay (in whole days) for each snakebite patient. The charts present the number of snakebite incidents in Cyprus from 2013 to 2019 by year of incidence and sex (male or female), as well as by age group and sex. However, the dataset neither shows the monthly number of admitted male and female snakebite victims, nor the monthly age group distribution, nor the length of stay of the monthly admitted patients. It also does not provide information of the length of stay separately for both sexes, nor for the age groups. The data were obtained from two small rural hospitals providing primary healthcare (Polis Chrysochous and Kyperounta), from four district general hospitals offering secondary healthcare (Paphos, Limassol, Famagusta and Larnaca) and from Nicosia General hospital, which provides tertiary healthcare [33]. Complex medical cases are referred from the rural hospitals in Polis and Kyperounta to the large general hospitals in Paphos and Limassol, respectively [34]. Population growth data (2001–2019) were obtained from the Statistical Service of Cyprus [35] and rainfall records (2013–2019) from the Department of Meteorology website [36]. To observe trends in the annual occurrence of snakebite in Cyprus, we calculated snakebite incidence (annual number of hospitalized persons due to snakebites per 100,000 inhabitants) from 2013 to 2019. Data on human-viper conflict were acquired from semi-structured interviews conducted by Daniel Jestrzemski (DJ) in the spring of 2014 with 12 representatives of Cypriot institutions related to the sectors of health, forestry, agriculture, environment and wildlife management. Of these, 10 interviewees provided information cited in this article (Table 1). The interviews took up to 60 minutes time and focused on aspects such as the public perception and management of *M. l. lebetina* and other Cypriot snake species, and on subject-related training by staff of the respective institutions. Interviews further included questions on the ecology, abundance, snakebite epidemiology and public perception of the blunt-nosed viper, its conservation status and possible future approaches to snake management and conservation in Cyprus. Additional questions were suited to the work of the respective institutions, providing the possibility for participants to include individual experiences. All participants expressed their consent for scientific evaluation of the provided information, and for the disclosure of their names. The information from the interviews were summarized in a MS Excel file, by dividing it into the categories “blunt-nosed viper habitat”, “blunt-nosed viper species trend on Cyprus”, “protection measures for blunt-nosed vipers”, “problems caused by blunt-nosed vipers on Cyprus”, “threats to the Cypriot blunt-nosed viper” and “recommended measures”.

**Table 1:** Representatives of Cypriot institutions interviewed on the conflict between people and blunt-nosed vipers in Cyprus

Institution	Representative	Date of interview
Troodos Visitor Centre (Troodos National Forest Park), Department of Forests, Ministry of Agriculture, Rural Development and Environment	K. Kailis	15 April 2014
Stavros Tis Psokas Forest Station (Paphos Forest), Department of Forests, Ministry of Agriculture, Rural Development and Environment	X. Ioanou	24 April 2014
District Agriculture Office (Polis Chrysochous), Department of Agriculture, Ministry of Agriculture, Rural Development and Environment	A. Pavlou	23 May 2014
Polis Chrysochous Hospital	M. Theodorou	27 May 2014
Department of Forests, Ministry of Agriculture, Rural Development and Environment	H. Nicolaou	28 May 2014
Department of Environment, Ministry of Agriculture, Rural Development and Environment	E. Erotokritou	28 May 2014
Gunsmith and hunting instructor from Skoulli village (Paphos district)	H. Demetriades	30 May 2014
Cyprus Game and Fauna Service	H. Hadjistyllis	6 June 2014
Cyprus Herpetological Society	H.-J. Wiedl, V. Schrempf	29 March 2014, 4 April 2014, 10 April 2014, 17 August 2014

## Results

### Snake envenomation in the Republic of Cyprus from 2013 to 2019

**Fig. 3:**
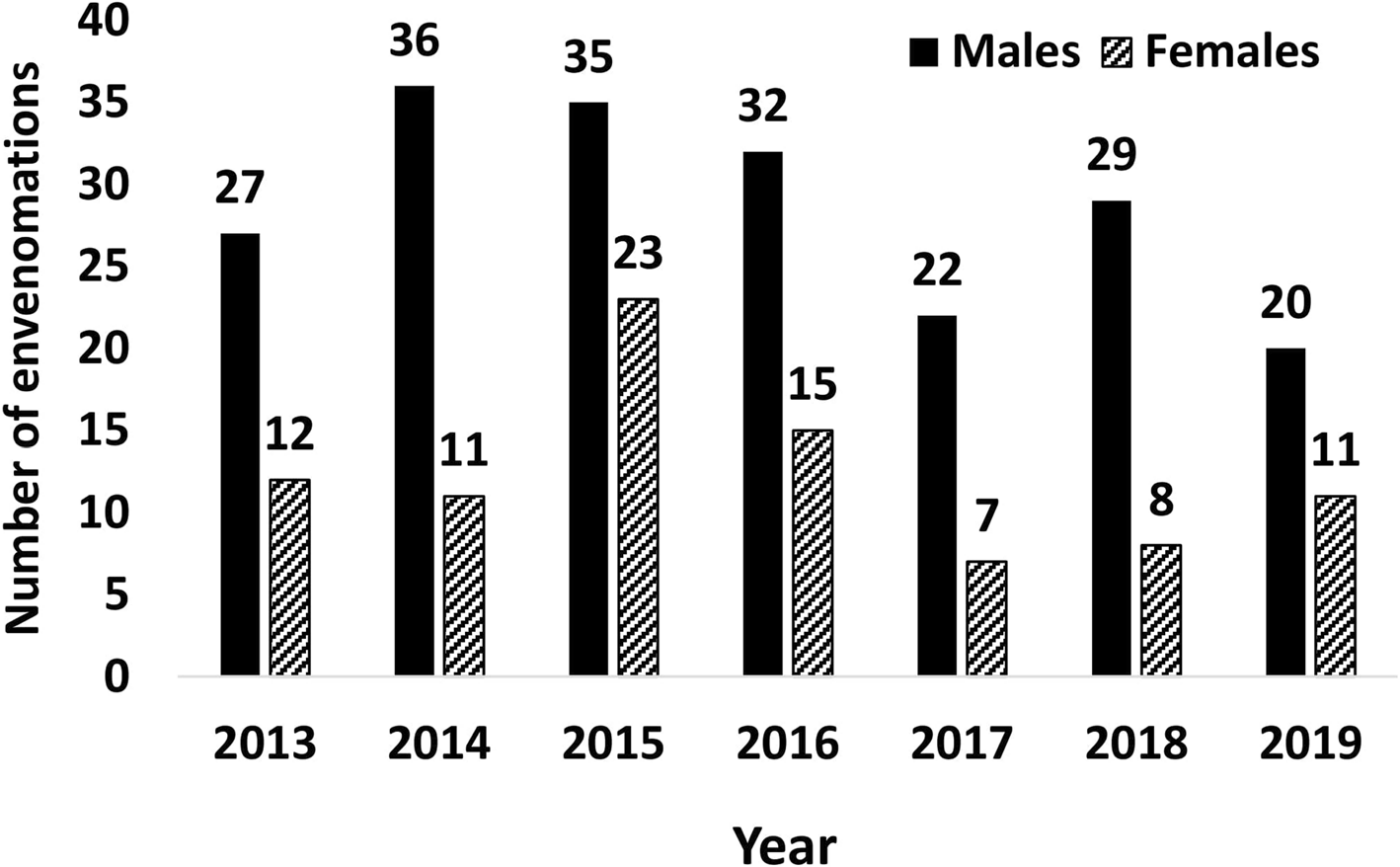
Snake envenomation in the Republic of Cyprus during the period from 2013 to 2019, by year of occurrence and sex

From 2013 to 2019, 288 cases of snake envenomation were recorded in the Republic of Cyprus. During the seven-year period, an average of 41 people were admitted to public hospitals due to snakebite annually, with a minimum of 29 bites in 2017 and a maximum of 58 in 2015. While most snakebites took place in the years 2014–2016 (47, 58 and 47 bites, respectively), the number was lower for the consecutive 3 years, with 29 bites occurring in 2017, 37 bites in 2018 and 31 bites in 2019 (Fig. 3). The annual incidence of admissions due to venomous snakebite per 100,000 people in the Republic of Cyprus rose from 4.55 (2013) and 5.55 (2014) to 6.84 (2015), but dropped to 5.50 in 2016. It remained on a lower level since then, with an incidence of 3.36 (2017), 4.22 (2018) and 3.49 (2019). The mean incidence of snakebite (2013–2019) was 4.79.

### Deaths related to snakebite from 2000 to 2019

Two deaths were linked to snakebite in Cyprus during the period from 2000 to 2019. In July 2004, a 73-year-old man died as a direct result of snake envenomation. Consequently, the underlying cause of death was coded X20 [31], referring to “contact with venomous snakes”. The second death occurred in April 2015 in an agricultural field and concerned a 77-year-old woman. Unlike the first case, it was related to multiple causes. These included cardiorespiratory arrest (I469, five hours), arterial hypertension (I10, 10 years), hyperlipidemia (E785, 10 years), postsurgical hypothyroidism (E890, 15 years), arteriosclerosis (I709, 15 years), overexertion (T733, X50), possible heatstroke (T670, X30) and the possibility of snakebite (T630, X20). According to the IRIS analysis, hyperlipidemia (unspecified, E785) was considered as the underlying cause of death, although snakebite could not be excluded as well. For both cases, no patient charts were available, and it is not known whether antivenom was administered.

### Length of stay in hospital

**Fig. 4:**
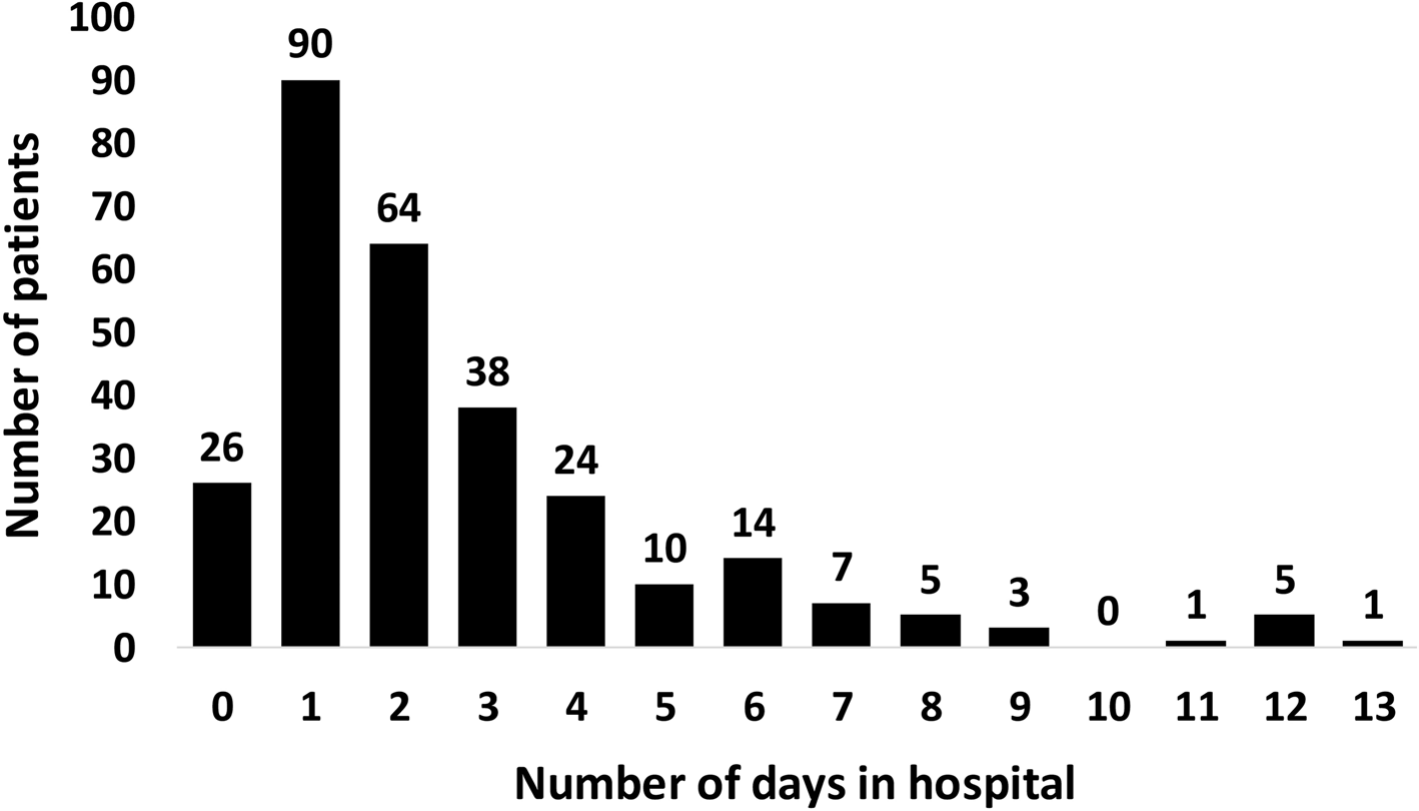
Number of days in hospital spent by 288 snakebite victims in the Republic of Cyprus (2013–2019)

From a total of 288 patients diagnosed with snake envenomation, 90 (31.25%) spent 1 day in hospital, followed by 64 patients (22.22%) with a 2-day stay, 38 patients (13.19%) who spent 3 days in hospital and 26 patients (9.03%) who were discharged on the same day (Fig. 4). Of all 288 patients, 242 (84.03%) were discharged after 4 days or less in hospital. Six people (2.08%) spent longer than 10 days in hospital, namely 11 days (one case), 12 days (five cases) and 13 days (one case). On average, snakebite patients spent 2.65 days in hospital, while the median was 2 days.

### Annual occurrence of admissions due to snake envenomation in Cyprus

**Fig. 5:**
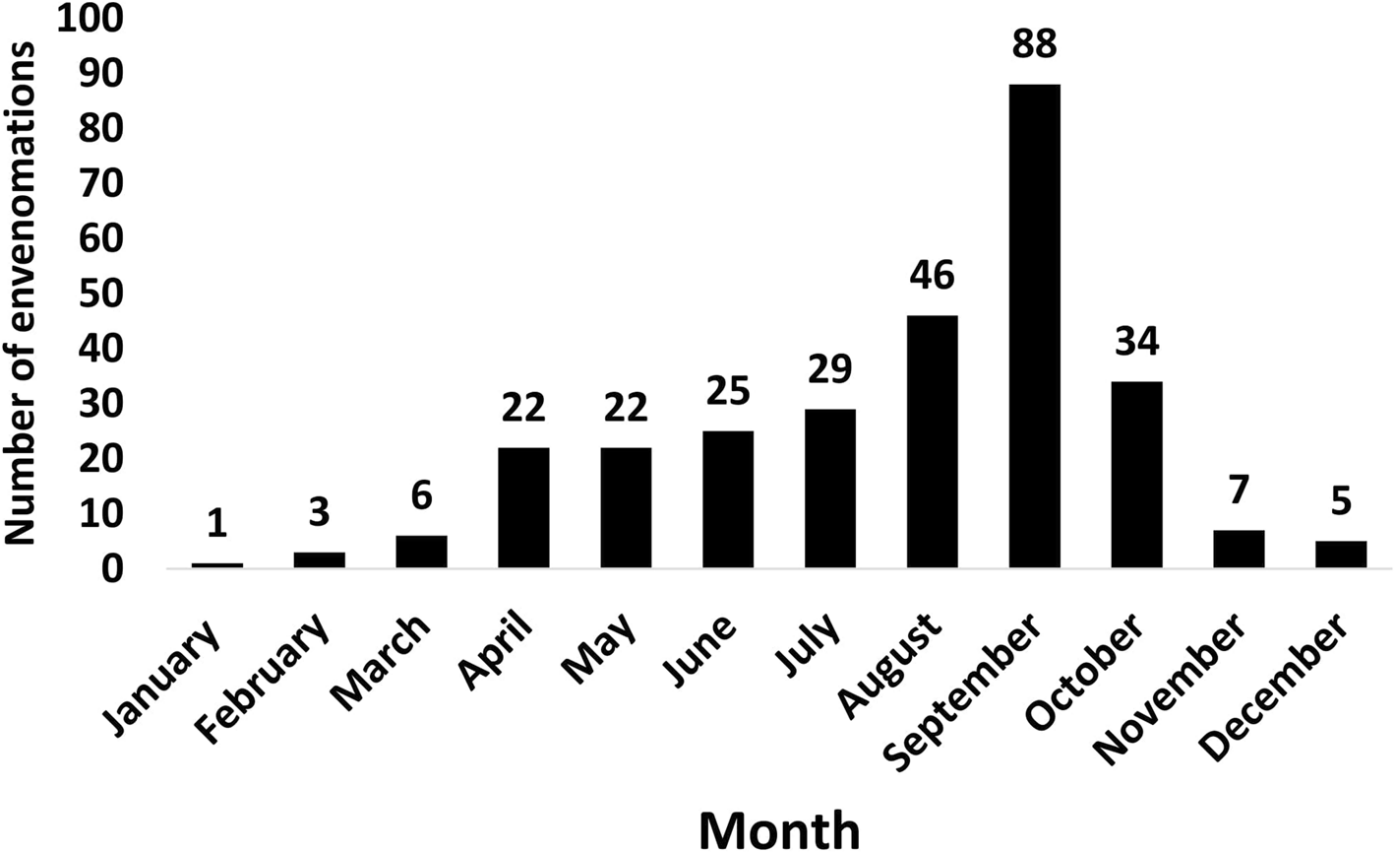
Snake envenomation in the Republic of Cyprus per month (cumulative for 2013–2019)

From winter (January to March) to spring (April to June), a steady increase in the average number of snake envenomation cases per month could be observed, culminating in late summer (September), and then decreasing towards autumn (October to December) and winter. Consequently, the average number of snake envenomation cases per month (2013–2019) ranged from 0.14 in January to 12.57 in September, whereas the monthly average for a whole year was 3.43. Of all 288 cases of envenomation, 266 (92.36%) took place between April and October (Fig. 5), which coincides with the activity period of *M. l. lebetina*. During the colder months (November to March), blunt-nosed vipers hibernate, while they are most active during the mating season (April to May), but also show activity during summer (June to September). Snake envenomation culminated during the dry summer season, with 188 cases (65.28%) occurring from June to September (2013–2019). During the peak of the dry season (August and September), 134 incidents (46.5%) took place. During the rainy winter and spring seasons (2013–2019), only 100 cases of envenomation (34.72%) were registered. On average, 27 persons per month were bitten during the dry season, and 14 persons per month during the rainy season. Over the year, most snake envenomations took place in September (88 cases in total).

### Distribution of sex and age groups among admitted snakebite victims in Cyprus

**Fig. 6:**
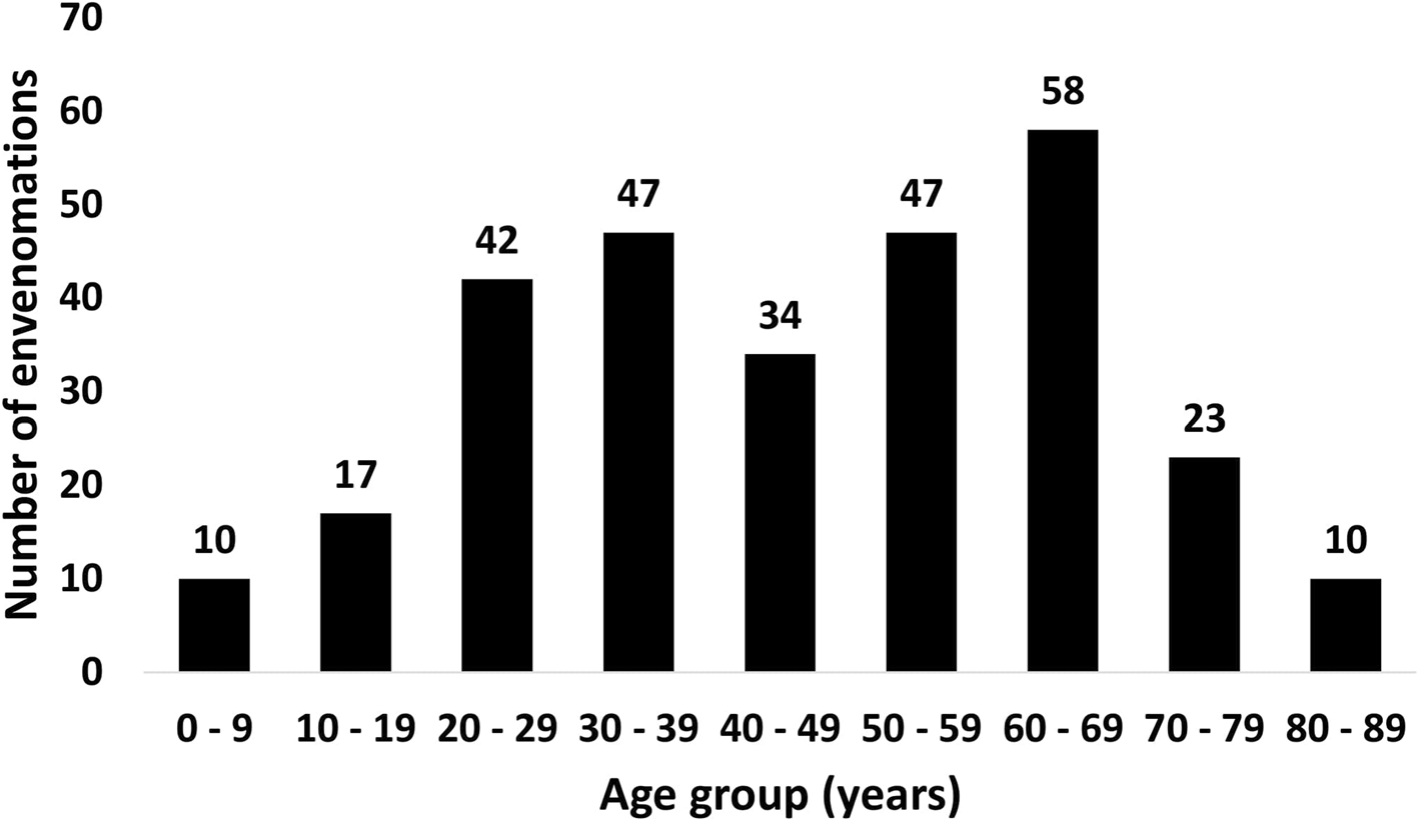
Snake envenomation in the Republic of Cyprus by age group (2013–2019)

Most patients admitted for snake envenomation were males (201 cases or 69.79%), compared to only 87 cases (30.21%) involving females. Correspondingly, of 41 people bitten on average every month (2013–2019), 29 (69.79%) were males and 12 (30.21%) were females. Hence, the average snakebite incidence in Cypriot males (6.85 per 100,000 population) was more than twice as high as the snakebite incidence in females (2.82). The snakebite incidence of males peaked in the years 2014 (8.74), 2015 (8.48) and 2016 (7.68), and was lowest in 2019 (4.60). In contrast, snakebite incidence in females peaked in 2015 (5.28) and was still above average in 2016 (3.42), before in 2017 dropping to its lowest value within 2013–2019 (1.58). The age of snakebite patients ranged from very young (0–4 years) to very old (85–89 years). Between 2013 and 2019, the youngest (0–9 years) and oldest age group (80–89 years) were each represented with 10 recorded cases, and 1.43 cases of snake envenomation per year on average. Middle and older age groups were most heavily affected, with 47 cases each in the age groups of 30–39 and 50–59 years (6.71 cases of snake envenomation on annual average), and 58 cases in the age group of 60–69 years (8.29 on annual average). Hence, by far the highest mean snakebite incidence for the period of 2013–2019 occurred in the age group of 60–69 years (9.19 per 100,000 population), followed by the age groups of 50–59 (6.24) and 70–79 (5.44). The age group of 80–89 years (4.98 per 100,000 population) was almost equally affected as the age group 30–39 years (5.06), while incidence was lower in the age groups of 40–49 years (4.35) and 20–29 years (4.33). By far the lowest incidence was observed among 0–9-year-olds (1.50) and 10–19-year-olds (2.53) (Fig. 6).

### Distribution of snakebite-related hospital admissions among major hospitals in Cyprus

**Fig. 7:**
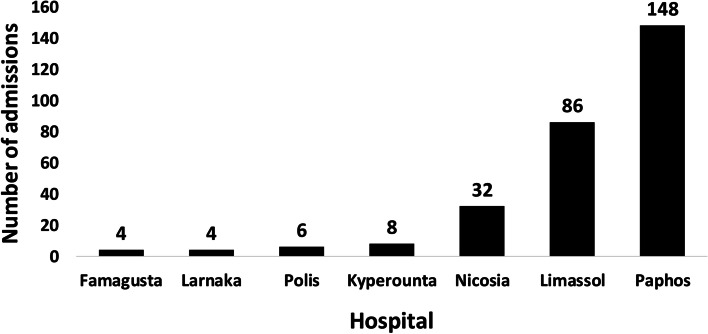
Number of snakebite admissions in the Republic of Cyprus by hospital (2013–2019)

Of all 288 hospital admissions related to snake envenomation between 2013 and 2019, 148 cases (51.39%) were recorded at Paphos General Hospital, 86 cases (29.86%) at Limassol General Hospital and 32 cases (11.11%) at Nicosia General Hospital. Another eight (2.78%) and six (2.08%) cases were treated at Kyperounta and Polis Rural Hospital. Ammochostos (Famagusta) and Larnaca General Hospital each reported four cases of snakebite (1.39%) (Fig. 7).

While approximately only one case of envenomation per year was recorded on average at the hospitals in Kyperounta (1.14), Polis (0.86), Ammochostos (Famagusta) and Larnaca (each 0.57), the number of envenomation cases per year was greater for the hospitals in Nicosia (4.57) and Limassol (12.29), and by far the highest at Paphos General Hospital, with an annual average of 21.14 snake envenomations for the period from 2013 to 2019. The cumulative number of snake envenomations per month and hospital was highest for Paphos Hospital too, where a total of 47 envenomation cases were registered in September (2013–2019). In addition, more than half of all envenomations affecting females (51 cases or 58.62%) as well as 48.26% of all envenomed males (97 cases) were recorded at Paphos Hospital.

### Factors increasing the risk of snake envenomation in Cyprus

In Cyprus, local people are usually afraid of *M. l. lebetina*, and often try to kill it on sight. Rear-fanged venomous snake species (e.g., *M. insignitus*), if mistaken for vipers, are persecuted as well (V. Schrempf, H.-J. Wiedl, pers. comm., 29 March 2014; E. Erotokritou, H. Nicolaou, pers. comm., 28 May 2014; H. Demetriades, pers. comm., 30 May 2014; H. Hadjistyllis, pers. comm., 6 June 2014). For many people, the adult, black stage of the large whipsnake (*Dolichophis jugularis*) is the only snake they do not kill, since they can clearly distinguish it from *M. l. lebetina* ([37]; H. Hadjistyllis, pers. comm., 6 June 2014). People working outdoors (e.g., farmers, forestry employees, game wardens, hunters, mosquito control workers and shepherds) encounter vipers more often than those from other occupational groups, thus being constantly exposed to the risk of snake envenomation [10]. In spring, blunt-nosed vipers commonly ambush birds and rodents from underneath shrubs, including caper (*Capparis spinosa*). Local people harvesting the popular fruits (see Fig. 2C) thus face an increased risk of blunt-nosed viper bites ([38]; H.-J. Wiedl, pers. comm., 29 March 2014). Shepherds persecute vipers to protect themselves as well as their livestock and dogs from snakebite, thus getting into contact with vipers as well (H.-J. Wiedl, pers. comm., 29 March 2014; V. Schrempf, pers. comm., 10 April 2014; H. Hadjistyllis, pers. comm., 6 June 2014). The killing of vipers by farmers and shepherds is also economically motivated, since the veterinary costs of treating a bitten, envenomed domestic animal often exceed the animal’s market value (H. Hadjistyllis, pers. comm., 6 June 2014). In Cyprus, hunting is a very popular sports activity in which about 25% of the male population are engaged [2], and hunters commonly get into contact with vipers. They are not only afraid of being bitten themselves, but also want to protect their hunting dogs from snakebite (V. Schrempf, H.-J. Wiedl, pers. comm., 29 March 2014; K. Kailis, pers. comm., 15 April 2014; H. Demetriades, pers. comm., 30 May 2014; H. Hadjistyllis, pers. comm., 6 June 2014). As a hunting dog in Cyprus may cost about 2000 € on average (about 6–10 times as much as a goat or sheep), hunters have a particular economic interest in keeping vipers away from their dogs (H. Hadjistyllis, pers. comm., 6 June 2014). Farmers often kill blunt-nosed vipers which they find on their lands, in contrast to the large whipsnake, which they usually accept (K. Kailis, pers. comm., 15 April 2014; A. Pavlou, pers. comm., 23 May 2014). Sometimes farmers use nets to catch and kill blunt-nosed vipers. In one case, 17 vipers were captured in a single net within few months (H. Hadjistyllis, pers. comm., 6 June 2014). Mosquito control workers employed by the Health Department enter streams all over Cyprus on a daily basis, thus permanently encroaching on habitats of *M. l. lebetina* [10]. Each mosquito control worker may encounter dozens, and even more than 100 blunt-nosed vipers over the course of a year. They regard vipers as a hazard and commonly kill them (V. Schrempf, pers. comm., 29 March 2014; H.-J. Wiedl, pers. comm., 4 April 2014; H. Demetriades, pers. comm., 30 May 2014; H. Hadjistyllis, pers. comm., 6 June 2014). So far, there are no educational programs on snakebite management for mosquito control workers in Cyprus (H. Demetriades, pers. comm., 30 May 2014), which exacerbates the risk of snakebite faced by this occupational group. Forestry employees frequently encounter and kill vipers, too, although they are instructed to stay away from snakes (K. Kailis, H.-J. Wiedl, pers. comm., 15 April 2014; H. Nicolaou, pers. comm., 28 May 2014; V. Schrempf, pers. comm., 17 August 2014). Workers of the Cyprus Game and Fauna Service regard vipers as a threat to birds and kill them, particularly when they see them next to bird cages or water pools established for wildlife (H. Hadjistyllis, pers. comm., 6 June 2014). The ongoing transformation of wild viper habitat into real estate due to property development contributes to higher encounter rates between people and blunt-nosed vipers in Cyprus, thereby increasing the risk of snakebite on the island. Wildfires of anthropogenic origin take place every year and contribute to the loss of wild snake habitat, thus driving snakes closer to people [10]. Another major risk factor are gardens, agricultural areas and other man-made landscapes with ideal conditions for rodent populations (e.g., organic waste), which in turn attract blunt-nosed vipers ([38]; H.-J. Wiedl, pers. comm., 29 March 2014). The additional presence of wooden debris in gardens and agricultural landscapes provides for suitable snake hiding places. These structures enable vipers to live close to human settlements, which strongly increases the risk of snakebite in Cyprus [10]. Finally, due to the widespread public aversion to *M. l. lebetina*, it is the only snake species that is not protected in the Republic of Cyprus [2], which further increases the risk of persecution by humans, and thus, the snake envenomation risk in Cyprus.

## Discussion

### Snakebite incidence in the years 2013–2019

Like the absolute number of snakebites, snakebite incidence peaked in the years 2014 (5.55 per 100,000 population), 2015 (6.84) and 2016 (5.50). This temporary increase took place during a slight reduction in the population of the Republic of Cyprus, which was lower between 2014 and 2016 (850,033 on average) compared to the 2013–2019 average of 862,314. At the same time, the mean annual precipitation (2014–2016: 435.6 mm) was lower than the mean precipitation for the 2013–2019 period (475.8 mm). This appears surprising, as rainfall is usually positively correlated with the reproductive activity of venomous snakes, and is of critical importance for agriculture [30]. Consequently, wetter years might have been expected to result in increased farming activities combined with prolonged movements of snakes, resulting in higher encounter rates between people and venomous snakes. The data of the study period suggest that this was not the case in Cyprus: Although the precipitation in 2018 (606.5 mm) and 2019 (796.8 mm) was 27% (2018) and even 67% (2019) above the annual average from 2013 to 2019, snakebite incidence was only 4.22 in 2018 and 3.49 in 2019, which was 12% (27%) below the annual average from 2013 to 2019 (4.79).

It is also noteworthy that the wettest year of the 7-year period (2019) had the second lowest snakebite incidence. However, most of the rainfall of 2019 occurred in December (208.3 mm), a month with generally low levels of snakebite. Still, precipitation during the dry season from June to September was much higher in 2018 (41 mm) and 2019 (43.4 mm) than in 2014 (34.5 mm), 2015 (13.3 mm) and 2016 (12.7 mm), although less snakebites were recorded during the dry season in 2018 (26) and 2019 (21) than during the same period in 2014 (33), 2015 (35) and 2016 (30). Collectively, this suggests the possibility that the more widely distributed availability of water in the summers of those years that received unusually high precipitation might have resulted in a reduced need for snakes (and perhaps also humans) to assemble near the usually few remaining bodies of water, which in turn might have resulted in less frequent encounters between snakes and humans.

### Snakebite occurrence over the year

The culmination of snakebite cases in Cyprus during the dry summer season is in line with the frequent observation of *M. l. lebetina* close to water during this period. As the Cypriot summer proceeds and landscapes dry out, water bodies provide significant advantages for blunt-nosed vipers such as shelter, ambush sites, ease of water uptake, and thermoregulation. Consequently, there is a constant risk for people to encounter *M. l. lebetina* at water bodies (including swimming pools) during summer [10]. Although blunt-nosed vipers usually hibernate from November to March in Cyprus, they may still be active in winter under favorable weather conditions [2]. This can explain the 22 cases of envenomation recorded between November and March (2013–2019).

### Sex ratio, age distribution, length of stay in hospital and snakebite mortality

The highly unequal distribution of snake envenomation incidence among sexes (6.85 per 100,000 population in Cypriot males compared to 2.82 per 100,000 population in Cypriot females) underlines the increased risk of bites by venomous snakes that males face compared to females in Cyprus. An explanation might be that in Cyprus, occupational outdoor activities are possibly more commonly pursued by men, who thus may be more likely to encounter *M. l. lebetina*. This sex-specific bias is also found in tropical developing countries, where snakebite is considered an occupational disease most commonly affecting young agricultural workers, particularly men [39, 40]. The presence of very young and old age groups as well as women in the snakebite statistics indicates that not only middle-aged people and men are at risk of venomous snakebite on the island. In fact, snakebites in Cyprus also take place in private gardens, where they are more likely to affect people of both sexes and all age groups alike [10]. People of 50 years and older made up 48% of all registered snakebite victims, although accounting for only 32% (2013), 33% (2014, 2015) and 34% (2016–2019) of the Cypriot population, which hints at an increased risk of snakebite faced by these parts of the population. One reason might be the higher representation of older age groups (50+ years) in rural areas with nearby blunt-nosed viper populations. However, on the one hand, the proportion of these age groups did not differ strongly between the rural (32.5%) and urban (30.3%) population of Cyprus in 2011 [41]. On the other hand, there were remarkable differences between the districts of Paphos, Limassol, Nicosia and Larnaca in the proportion of older age groups (50+ years) among the rural and urban populations. While the proportion of 50+ year-old people was almost equally represented in rural and urban areas of Nicosia (30.1% vs 31.0%) and Larnaca district (30.4% vs 29.5%), 50+ year-old people made up a higher proportion in the rural population of Limassol district (35.1% vs 30.3% urban), and were especially dominant among the rural population in Paphos district (45.7% vs 28.8% urban) [41]. Thus, older-aged people in Paphos district are more likely to encounter venomous snakes than in other districts. Interestingly, Paphos is also the district with the highest observed abundance of *M. l. lebetina* in Cyprus [2], which may contribute to generally higher snake envenomation rates in this district. Furthermore, it can be assumed that traditional outdoor activities (e.g., shepherding, farmwork) are more commonly pursued by older-aged people (own observations), which additionally increases their risk of encountering blunt-nosed vipers. This assumption is supported by the strong representation of the 50–69-year-old, who account for 76% of all snakebite victims over 50, and who are younger and thus more likely to conduct physically demanding outdoor activities than people of 70–89 years of age. With approximately 20% of all envenomations, the age group of 60–69 years is most strongly affected, which could be due to less effective prophylactic measures undertaken against snakebite by people within this age group. An explanation may be that older people in rural areas apply modern outdoor safety measures (e.g., wearing safety gloves and protective boots for farm work) less commonly than younger people, as older persons may show less risk aversion than their younger counterparts [42]. The fact that 84% of all patients spent a relatively short time in hospital (up to 4 days) suggests clinical courses of snake envenomation in Cyprus that are mostly mild and/or uncomplicated. This is further highlighted by the low average number of days spent by snakebite patients in hospital (2.65). However, the median (2 days) would be a more appropriate measure, as most patients (62.5%) stayed in hospital for a maximum of 2 days only, while the relatively small share of patients with a stay of 5 days or more (16%) is responsible for the strong skewness of the distribution to the left. With one directly and another indirectly related death in 288 hospital admitted snakebite cases between 2013 and 2019, the snakebite mortality in the Republic of Cyprus was very low.

### Distribution over hospitals

**Fig. 8:**
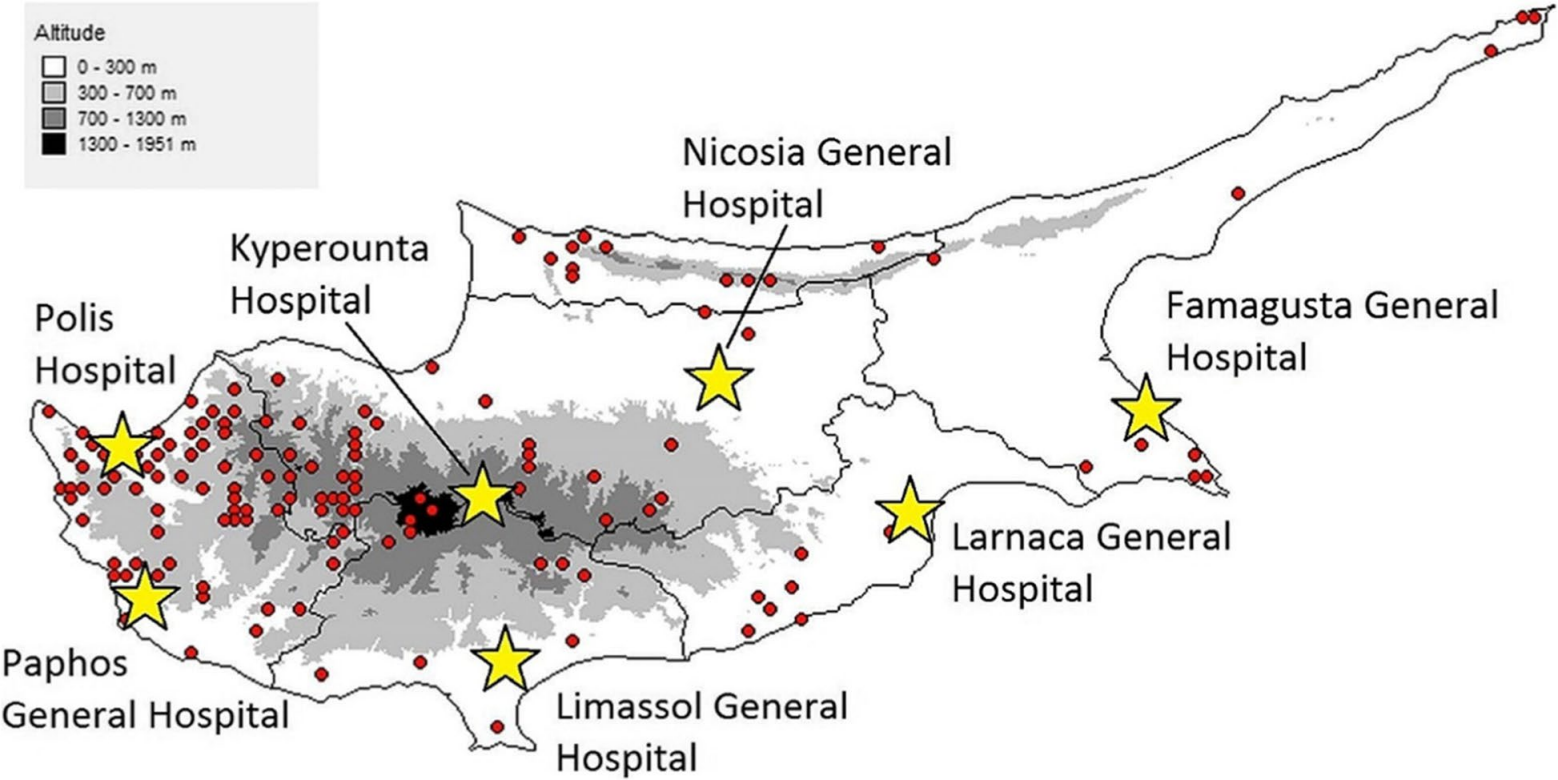
Distribution of *Macrovipera lebetina* in Cyprus, shown by red dots (map from Baier et al. 2013 [2]). The location of the seven hospitals in the Republic of Cyprus is shown by yellow stars

The numbers of snakebite-related public hospital admissions in four major Cypriot district hospitals (2013–2019) do not correlate with the population sizes (2019) of the corresponding districts. While the population size of Paphos district is 95,400 (only 11% of the population of all five districts), 51% of all hospital-admitted snakebite cases in the Republic of Cyprus were registered at Paphos General Hospital alone. On the other hand, Larnaca and Famagusta, with a combined total of 197,900 inhabitants (22% of the population) only had eight cases (3%) of snake envenomation at their two major district hospitals. With 346,400 inhabitants, Nicosia district has a share of 39% of the population, but only 32 people (11% of all cases) were admitted with snake envenomation to the district’s general hospital from 2013 to 2019. In comparison, the recording of 30% of all snakebites (86 cases) at Limassol General Hospital is approximately proportional to the number of Limassol district residents (248,300), which amounts to 28% of the population of all five districts. The registration of slightly more than half of all hospital-admitted snakebite cases only at Paphos General Hospital is notable, as Paphos is also the district with the highest density of *M. l. lebetina* in Cyprus (see Fig. 8). While the common occurrence of *M. l. lebetina* in Paphos may hint at an increased risk of snake envenomation in this district, it does not necessarily have to correlate with the number of admissions to Paphos General Hospital, as snakebite victims may be transferred from other districts as well. Connectivity may be an important factor, as Paphos General Hospital can be easily reached from Limassol via the A6 Highway, and from Polis via the B7 country road, and is situated less than 500 m away from the main junction of both roads. Also, as the five district general hospitals are better equipped to manage cases of snake envenomation, snakebite victims may be referred to them from rural hospitals, or directly seek care at secondary or tertiary referral centers. For example, people bitten in the Polis Chrysochous area may prefer to visit Paphos General Hospital, instead of presenting to the nearby hospital in Polis. In fact, the snakebite-related admission numbers in the rural hospitals of Polis and Kyperounta (5% of all cases) are surprisingly low, given that both are situated in areas with common occurrence of *M. l. lebetina* (see Fig. 8), which hints at the above-mentioned trend. Finally, a possible explanation for the outstandingly high number of snakebite-related admissions to the general hospitals in Paphos and Limassol (together accounting for 81% of all admissions) may be the double-counting of patients, due to the transfer of inpatients from other hospitals (e.g., in Polis). In such a case, a snakebite patient being transferred between two different hospitals will be registered as two different envenomation cases. As the available dataset does not provide the patients’ personal information, there is no mechanism to avoid double-counting, and hence the patient can be easily confused with two different persons (each at another hospital). This may lead to particularly high numbers of registered patients in larger hospitals.

### Care-seeking behavior of snakebite victims

Since the available dataset of this retrospective study does not include information on the care-seeking behavior of snakebite victims, there is no empirical basis for identifying and discussing the factors that determine their hospital selection preferences (e.g., accessibility, reputation, cost, specialization, superior equipment, number of available beds). However, public hospitals in Cyprus are free of charge for all patients presenting to the emergency department and are also much less costly for in-patients than private hospitals. Hence, these circumstances suggest that at least snakebite victims with a low-to-average income would preferably visit public hospitals. However, no data were available from private hospitals, for inpatients.

### Antivenom administration in Cyprus

Since at least 1971, antivenom is given to documented victims of snake envenomation in Cyprus**,** who present to the emergency department of public hospitals. “Polyvalent Snake Venom Antiserum” produced by the Egyptian Organization for Biological Products & Vaccines (VACSERA, Cairo, Egypt) has been used since at least 2009 to treat snake envenomation in Cyprus (M. Antoniou [Pharmacist at Nicosia General Hospital], pers. comm., 5 November 2020). The polyvalent antivenom from VACSERA is raised against the venoms of several venomous snake species, specifically from *Naja haje, N. nigricollis* and *Cerastes cerastes* [43], although its efficiency has not yet been tested by the WHO [44]. According to the manufacturer, it also has paraspecific neutralizing activity against the venoms of several oriental viper species, including *M. lebetina* [45]. As several antivenoms exist that are raised against the venoms of palaearctic vipers [44, 46], in some cases even specifically against the venoms of *M. lebetina* subspecies (i.e., monospecific antivenom against *M. l. turanica* by UzbioPharm (Tashkent, Uzbekistan) [44, 47]), it may be expected that the latter have better neutralizing capacities against *M. l. lebetina* venoms. Besides the monovalent antivenom from UzbioPharm, polyvalent antivenoms with stated (specific or paraspecific) neutralizing activity against *M. lebetina* venoms are produced by different manufacturers in addition to VACSERA, including the Institut Pasteur de Tunis (Tunis, Tunisia), the Institut Pasteur d’Algerie (Algiers, Algeria), the Imunološki Zavod (Institute of Immunology, Zagreb, Croatia), the Institute of Virology, Vaccine and Sera TORLAK (Belgrade, Serbia), Inosan Biopharma S.A. (Madrid, Spain), the Razi Vaccine & Serum Research Institute and the Padra Serum Alborz (both in Karaj, Iran), as well as the Vetal Serum ve Biyolojik Ürünler Üretim Sanayi Tic. A. Ş. (Adıyaman, Turkey) [44]. The WHO (2016) furthermore lists the National Institute of Health in Islamabad (Pakistan) among the producers of polyvalent antivenom against *M. lebetina* venom [48].

### Medical importance of *M. lebetina*

Although *M. lebetina* has been rated as one of the snake species of highest medical importance (WHO category 1) in Algeria, Iraq, Iran, Lebanon, Syria, Turkey, Armenia, Azerbaijan, Georgia, Kazakhstan, Kyrgyzstan, Tajikistan, Uzbekistan, Turkmenistan and Afghanistan, and as a snake species of secondary medical importance (WHO category 2) in Cyprus, Jordan, Tunisia and Pakistan [49], there clearly is a lack of clinical and epidemiological research on envenomation caused by these widely distributed vipers. While a few published case reports illustrate the range of symptoms and signs that may follow envenomation by this species, most are anecdotal or offer little clinical detail [2, 10, 29, 50]. Fraser [12], Hopkins [51] and Göçmen [52] described bites caused by *M. l. lebetina* from Cyprus. In the first case, occurring 1929, a shepherd was bitten on the scalp while kneeling down to drink from a forest pool in Cyprus. He was admitted to the hospital in Famagusta (8 h after the bite), and discharged 11 days later. This patient initially suffered from a loss of consciousness, massive edema and partial loss of speech, and his bite and incision wounds healed slowly [12]. Two other bites described by Hopkins (1974) occurred when army personnel from the United Nations Peacekeeping Force in Cyprus (UNFICYP) were bitten on the back of the right hand (first case) and on the calf (second case). In the first case, there was initially swelling and vomiting, followed on the third day by high fever and signs of cellulitis. On the 15th day, the patient reported anesthesia and paralysis of his right arm almost up to his elbow. Paralysis resolved within 5 days and anesthesia within 26 days. In the other case, the patient initially suffered from severe anxiousness and cramps in the injured limb, but was almost free of symptoms on the following day [51]. A particularly severe case was reported by Schweiger (1983) who was bitten by a large (150–160 cm) *M. l. obtusa* in his right forearm near İskenderun (Turkey) and subsequently suffered massive painful edema of the bitten arm extending to the trunk and other arm, blistering, myonecrosis requiring the amputation of the bitten limb, thrombocytopenia, coagulopathy, haemorrhage, hypovolaemic shock, acute kidney injury, necrosis of the lungs, paraesthesia, complete loss of motor function of the legs, and contractures of the knee joints. Ten months after the bite, the patient had largely regained his ability to walk, albeit with highly reduced leg strength [53]. Göçmen (2006) reported a bite on a 40-year-old male researcher who was bitten into a right-hand finger by a 75-cm-long male Cypriot *M. l. lebetina*. Five minutes after the bite, edema started to develop and within 3 h advanced to the center of the right arm, accompanied by severe pain. Further symptoms included hypotension, shock, tissue necrosis, hemorrhage, melanoderma, and considerable quantitative differences in the fractions of albumin, globulin and albumin/globulin ratios. After 24 hours, the patient’s condition had normalized, and he was discharged from hospital. Complete recovery took place after the sixth week [52]. Sharma et al. (2008) described a case from Jammu and Kashmir, in which a 33-year-old male soldier resting in his bed was bitten in his left hand by a 94.5 cm long *M. l. cernovi*. Twelve days after the bite, the bitten finger had become necrotic and was amputated [54]. In another case in Kashmir, a 46-year-old farmer suffered a *M. lebetina* bite to his penis while urinating on an open field [55]. In both cases, the main symptoms of envenomation included edema, bruising and necrosis around the bite site. The second case in addition had mild coagulopathy but overall a milder clinical course and was discharged 36 hours after the start of treatment. Kazemi et al. (2019) presented five cases of snake envenomation by *M. l. obtusa* in Northwestern Iran, which occurred in spring 2019 and resulted in severe clinical courses and long-term musculoskeletal disabilities in some of the bite survivors. In three cases, a fasciotomy had to be performed, affecting the arm of a 15-year-old boy, the hand of a 36-year-old man, and the leg of another, 30-year-old man. In the latter case, extensive necrosis led to the amputation of the leg at the knee. All five patients received polyvalent anti-snake venom from the Razi Vaccine and Serum Research Institute, Karaj, Iran [56]. Cattaneo (2020) provides the first literature record of a bite incident by *M. l. schweizeri*. The victim, a young woman bitten on the right hand, was transferred to the hospital, where a sodium bicarbonate therapy was applied, coupled with the admission of drugs helping to replenish electrolytes and body fluids. The bitten limb swelled to the armpit, accompanied by pain, increased blood sugar and platelets, a reduction in red blood cells, minor hypotension and a decreased heart rate. The patient was discharged after 2 days, although the swelling and pain in the affected limb needed some additional days to dissolve [57]. Finally, Abukamar et al. (2022) reported of an envenomation by *M. l. obtusa* in Jordan. A 36-year-old male farmer bitten on his left foot presented to his local hospital, with initial symptoms such as dizziness and burning pain in the left leg. His bitten foot and ankle were swollen and hot, with extensive ecchymosis at the bite site. Despite treatment with antivenom, intravenous fluids and antibodies, his condition worsened. Even after transfer to a tertiary hospital in Amman, his platelet count continued to drop. Although neutrophilic leukocytosis and normochromic normocytic anemia were diagnosed, the patient finally recovered, and was discharged after 5 weeks [58].

### Recommendations for snake envenomation prevention

Since the risk of snake envenomation is a primary concern in Cyprus, educational workshops for occupational outdoor groups such as mosquito control workers, hunters, shepherds, farmers, forestry employees and game wardens are critical for raising awareness of the risks and prevention of snake envenomation. Workshops should also be offered to rural communities and schools and include the promotion of non-lethal methods for preventing human-viper conflict such as snake deterrence or translocation. The risk of encounters between people and *M. l. lebetina* in Cyprus can be further reduced by legally protecting valuable snake habitats such as wild riparian areas and rocky slopes with confirmed *M. l. lebetina* populations, with prohibition of land transformation and strict regulations concerning further anthropogenic interventions (e.g., grazing). This could be achieved by designating new areas for the Natura 2000 network of the European Union. In all conservation areas, hunting should be prohibited.

Finally, a major contribution towards reducing the risk of snake envenomation in agricultural landscapes and gardens is the removal of organic waste and other structures attracting rats, and of wooden debris and other elements serving as potential snake shelters (H.-J. Wiedl, pers. comm., 29 March 2014).

### Limitations of the study and suggestions for research protocol improvement

While the collection of data on annual hospital admissions due to snake envenomation in the Republic of Cyprus is a novel step towards a better understanding of the island’s snakebite epidemiology, the research protocol for further studies should include additional information, which will allow for a more precise snakebite data analysis. Since all records of hospital-admitted patients were based on the ICD-10 code T63.0, other ICD-10 codes related to snake envenomation (i.e., X20, contact with venomous snakes and lizards) were generally not listed by the hospitals, with exception of the two fatal cases, where the underlying causes of death were coded X20. However, X20 would be highly suitable for the non-fatal cases as well, as it explicitly includes vipers. Additionally, the applied ICD-10 code T63.0 can be split into four subcategories: accidental (001), intentional self-harm (002), assault (003) and undetermined (004) [59]. Although this sub-division was not provided for the dataset of this study, it can be assumed that all of the registered envenomations on Cyprus occurred accidentally (T63.001), as no case of assault or intentional self-harm (i.e., attempted suicide) involving snakes has – to the best of our knowledge – ever been published for Cyprus. Although this article provides for the first time systematically collected data on snakebite epidemiology in the Republic of Cyprus, it is doubtful that all cases of snake envenomation between 2013 and 2019 were included. This point can be illustrated by one incident in which an author of this article (DJ) was bitten on the back of his hand while handling a large male *M. l. lebetina* in Polis (Paphos district) on 29 May 2014. Although the bite was delivered into a safety glove, one fang penetrated the glove and punctured the skin. The bitten herpetologist was subsequently transferred to Polis Rural Hospital where he was given injections of cortisone (Solu-Cortef) and antihistamines (Phenergan). As no signs of envenomation could be detected, he was discharged after treatment. This bite accident, consequently, is not included in the 2014 record of ICD-10-coded diagnosis for Polis Rural Hospital. In fact, no case of snake envenomation was recorded in Polis Hospital during that year. Hence, future surveys on snakebite-related hospital admissions in Cyprus should also include incidents which might be considered as minor due to the absence of envenomation signs. Our dataset also does not show whether the 288 snakebite admissions refer to 288 or less persons. Accordingly, we cannot exclude the possibility that a person was bitten and admitted repeatedly to public hospitals, although the likelihood of such an occurrence can be considered as rather low. Yet, knowing the exact number of snakebite patients is critical for the calculation of incidence rates. As the recorded data do not include information on the distribution of age group and sex over month of admission and length of stay, it is not possible to investigate differences between seasonal peaks of snake envenomation in males and females in Cyprus, nor between the affected age groups over the year. It is further not possible to examine the data for a correlation between age group (and sex) and length of stay in hospital. In the same way, the missing information of the monthly distribution of length of stay prevents an analysis of possible seasonal trends in this regard. Protocols of future retrospective reviews could help to fill this gap by including this information. Additionally, the missing information on the localities where the bite accidents occurred currently prevents a statistic of snake envenomation in each district. As snakebites often take place in remote areas, people may travel large distances and cross borders to reach the nearest hospitals. Hence, to conclude from the frequency of snakebite-related admissions in a certain hospital to the presence of nearby areas with a possibly increased risk of snake envenomation, or with higher abundance of *M. l. lebetina*, is fraught with uncertainty. Therefore, recording the location of the accidents will help to identify snake envenomation hotspots and enable a snakebite statistic for each district. Furthermore, the snake species responsible for each bite should be recorded, if reliable identification is possible, in order to distinguish between bites by front-fanged and non-front-fanged snakes in Cyprus. This is necessary to determine whether a potential life-threatening envenomation (*M. lebetina*), a mild envenomation at most (*M. insignitus, T. fallax*), or no envenomation (any other snake species) has occurred. A correct snake species identification is also very helpful for a better assignment of clinical courses to the corresponding species. In the same way, the documentation of the major envenomation symptoms will facilitate distinguishing between potentially life-threatening and mild clinical courses, which is not currently possible with the data at hand. These points should be addressed by creating a more comprehensive, systematic reporting system for snake envenomation on Cyprus, which should include data of admissions from public as well as private hospitals. Finally, the establishment of a poison information center in Cyprus would be highly useful for a more comprehensive collection of data related to snake envenomation on the island.

## Conclusions

Although *M. l. lebetina* is a large viper with a large venom yield, and widely distributed in Cyprus, deaths from snakebite are very rare in the Republic of Cyprus. Over the year, the hazard of snakebite is highest in late summer, particularly during the peak of the dry season in August and September. Occupational outdoor groups (farmers, forestry employees, game wardens, hunters, mosquito control workers and shepherds) are probably at highest exposure of encountering *M. l. lebetina* and experiencing snake envenomation. There is further an increased risk in areas supporting rat populations, which in turn attract blunt-nosed vipers. Males are at higher risk of snakebite than females, as are middle- and older age groups in their 60s. Public hospital admission data (years 2013–2019) suggest that the risk of snakebite in the Republic of Cyprus is highest in Paphos district. Although the short average length of hospital stay suggests that the clinical courses of most snake envenomation cases may not be severe or complicated, further research is warranted to clarify questions regarding the clinical management of such patients. In order to raise awareness of the snakebite risk and how to prevent it, educational programs on snakebite prevention and first aid should be provided especially to outdoor occupational groups, rural communities and schools. Other prevention measures include an improved protection of wild snake habitats, and the removal of structures attracting rats and providing snake shelter in anthropogenic landscapes. Critical information, which is missing in the current dataset (e.g., the localities where the bite accidents occurred, the snake species responsible for the bites, major envenomation symptoms), should be documented in prospective studies in the future. The procurement of antivenoms to treat envenomation by *M. l. lebetina* in Cyprus should be guided by comparative pre-clinical safety and efficacy studies using Cypriot *M. l. lebetina* venoms and different commercially available candidate products, and clinical studies.

## Data Availability

The datasets generated and analyzed during this study are available from the corresponding author upon reasonable request.

## Abbreviations

E: Eastern longitude
N: Northern latitude
Fig.: Figure
